# Impact of screen use on behavior and sleep in patients with autism spectrum disorder

**DOI:** 10.1055/s-0045-1813641

**Published:** 2025-12-22

**Authors:** Matheus Eugênio de Sousa Lima, Lívia Maria Eugênio Lopes, Fernanda Eugênio de Sousa Lima

**Affiliations:** 1 Hospital de Saúde Mental Professor Frota Pinto, Núcleo de Atenção à Infância e Adolescência, Fortaleza CE, Brazil.; 2 Universidade de Fortaleza, Faculdade de Medicina, Fortaleza CE, Brazil.

**Keywords:** Autistic Disorder, Cell Phone Use, Internet Use, Sleep Wake Disorders

## Abstract

**Background:**

The increasing screen time among the pediatric population is a detrimental factor for cognitive and psychosocial development, especially for children with autism spectrum disorder (ASD). However, there are still many questions regarding its negative effects on behavior and sleep in this population group and, as of the writing of this study, there are no specific recommendations regarding screen use for children with ASD.

**Objective:**

To synthesize and analyze the current evidence on the association between screen exposure time, behavioral symptoms, and sleep disorders in ASD children.

**Methods:**

The authors provided a Preferred Reporting Items for Systematic Reviews and Meta-Analyses (PRISMA) checklist adapted review of studies that examined the association between screen time and both autistic symptoms and sleep disturbances in this patient population.

**Results:**

Research indicates that excessive screen use among ASD children, particularly in preschool-aged children, may be associated with significant behavioral, emotional, and sleep quality impacts. The screen time recommendations set by the World Health Organization for the general pediatric population could also be applied to these children, at least until new studies can clarify specific guidelines, taking their particularities into account.

**Conclusion:**

This review article explores the current evidence on the association between excessive screen time and both autistic symptoms and sleep disturbances in ASD, underscoring the relevance of further clinical investigation.

## INTRODUCTION

Autism spectrum disorder (ASD) is essentially defined as a neurodevelopmental disorder with heterogeneous manifestations, characterized by persistent deficits in social communication, as well as the presence of restricted or repetitive behaviors. 1 The latest revised version of the Diagnostic and Statistical Manual of Mental Disorders (DSM-5-TR) emphasizes the presence of limitations in socioemotional reciprocity, deficits in nonverbal behavior affecting social interactions, and difficulties in initiating, maintaining, and understanding interpersonal relationships. These characteristics must be present from early development and cause significant functional impairment in affected individuals. 2

Over the past years, an increasing prevalence of ASD has been observed, with studies indicating a rise from an estimated 1.1 to 2.3% in the pediatric population. However, this increase may also be attributed to factors such as modifications in the DSM-5 criteria, improved sensitivity of screening and diagnostic tools, greater ASD awareness among the general population and healthcare professionals, and enhanced access to healthcare services. 1 Considering that the global pediatric population comprises approximately 2 billion children aged 0 to 14 years, according to United Nations data, it is imperative that healthcare and education systems provide adequate support to families, ensure appropriate neurodevelopmental stimulation, and manage psychiatric comorbidities in ASD children. 3

From a behavioral and emotional standpoint, individuals with ASD may exhibit psychomotor agitation, irritability, self-injurious behaviors, and aggression toward others, in addition to being more susceptible to psychiatric comorbidities. 1 Among these, sleep disorders are particularly noteworthy, affecting 40 to 80% of ASD children. 4 Insomnia—especially difficulties in sleep initiation—circadian rhythm disturbances, nocturnal awakenings, restless sleep, and parasomnias are among the most frequently observed sleep disturbances in this population. 4 5

In this context, the increasing use of digital screens among pediatric populations has been recognized as a significant risk factor for cognitive and psychosocial development. 6 Given these concerns, the World Health Organization (WHO) recommends that children under 1-year-old should not be exposed to screens, children aged 2 to 4 years should have a maximum screen exposure of 1 hour per day, while children and adolescents aged 5 to 17 years should not exceed 2 hours of screen time per day. 7 However, to date, there are no specific recommendations for children with ASD.

The impact of screen use on individuals with ASD has been the subject of numerous studies, with evidence suggesting that these patients tend to have significantly higher screen exposure times compared to neurotypical children. This has been associated with severe developmental delays, worsening behavioral symptoms, and sleep disturbances. However, the existing findings remain somewhat conflicting. 8

Given the knowledge gaps in this field, the present article aims to synthesize and analyze the current evidence on the association between screen exposure time, behavioral symptoms, and sleep disorders in individuals diagnosed with ASD.

## METHODS

The present study is an integrative literature review, incorporating adapted elements from the Preferred Reporting Items for Systematic Reviews and Meta-Analyses (PRISMA) checklist. The study was conducted by selecting relevant academic-scientific databases, specifically Medline/PubMed and SciELO. The research process included the definition of descriptors based on the Health Sciences Descriptors (DeCS), the establishment of inclusion and exclusion criteria for article selection, the identification of studies meeting these criteria, and the presentation and analysis of the collected data. To minimize bias, two independent researchers conducted the search and selection of articles.

The inclusion criteria comprised studies published within the last 5 years, available online in English, Portuguese, or Spanish, and evaluating outcomes related to screen use in individuals with ASD. The exclusion criteria were narrative reviews, meta-analyses, and case reports, as well as studies that did not establish clear correlations between screen use and the symptoms addressed in the guiding research question: “What is the impact of screen use on behavioral symptoms or sleep disturbances in patients with ASD?”

The following search strategy was applied: “autism” AND “screen time”; “autism” AND “smartphone”. A total of 72 articles were initially identified, of which 44 were selected for further review based on titles and abstracts. After applying the inclusion and exclusion criteria, 10 studies were ultimately included in the final analysis.

The data analysis was conducted using the Participants, Intervention, Comparison, Outcomes, and Study design (PICOS) strategy. In the present study, the criteria were defined as follows: participants diagnosed with ASD up to 21-years-old, exhibiting excessive screen use (i.e., exceeding the recommendations established by the WHO), assessed in cross-sectional studies. Artificial intelligence tools, such as ChatGPT (OpenAI, Inc.), were used in the preparation of the manuscript for spelling corrections and translation into English.

## RESULTS

The findings from the selected studies are synthesized and presented in Table 1 . The reviewed studies included samples from six countries across three continents (Canada, China, United States, France, India, and Iran). The majority of participants were male, with a mean age ranging from 37 to 39 months (approximately 3-years-old). The measurement instruments varied among validated questionnaires, such as the Youth Screen Time Survey (YSTS), and study-specific tools designed to assess screen time and its characteristics. The data indicate that, in most studies, the average daily screen time exceeded 2 hours.

**Table 1:** Synthesis of the studies selected

Study	Country	Sample	Instruments	Screen time	Outcomes
Garcia et al., 2020 9	United States	49 ASD patients Mean age: 12.4 years; 78% boys	Self-developed questionnaire; ActiGraph GT9X	130 min/day	No significant relationship between screen time and sleep efficiency or sedentary behavior.
Samanta et al., 2019 10	India	100 ASD patients. Mean age: 4.33 years; 100% boys	CSHQ; self-developed questionnaire	Mean screen time not reported in the study. 60% of patients use screens for 2–3 h/day; 30% for >3; and 10% for <2	Greater screen use was associated with delayed sleep onset, anxiety, nocturnal awakenings, daytime sleepiness, and worsening CSHQ scores.
Cardy et al., 2021 11	Canada	127 ASD patients. Mean age: 11.7 years; 22% boys; 78% girls	Self-developed questionnaire	6.9 h/week (59.14 min/day)	Excessive screen use was associated with negative impacts on quality of life and mental health, particularly in girls; reduced social interaction in ASD children.
Dong et al., 2021a 12	China	193 ASD patients. Mean age: 37.39 months; 81.34% boys; 18.66% girls	Self-developed questionnaire; CARS; GDS-C	2.64 h/day	Screen use was associated with reduced language development and increased severity of ASD symptoms.
Dong et al., 2021b 13	China	101 ASD patients. Mean age: 39.33 months; 77.2% boys; 22.8% girls	Self-developed questionnaire; CARS; GDS-C	3.34 h/day	Excessive screen use correlated with increased severity of ASD symptoms and negative impacts on motor, adaptive, and social development.
Dong et al., 2023 14	China	358 ASD patients. Mean age: 42.20 months; 78.2% boys; 21.8% girls	GDS-C; CSHQ; CBCL	1.58 h/day	Screen use before bedtime was associated with behavioral issues, sleep disturbances, and somatic complaints.
Berard et al., 2022 15	France	249 ASD patients. Mean age: 9.1 years; 80.3% boys, 19.7% girls	Self-developed questionnaire; V ABS-II; ADOS-2; CBCL	Mean screen time not reported in the study. 37.4% exceed the screen time limit (≤1 h/day for <5-year-olds; ≥2 h/day for >5-year-olds)	Excessive screen time was associated with reduced social interaction and abilities of daily life, especially in adolescents.
Sadeghi et al., 2023 16	Iran	68 ASD patients. Mean age: 27.09 months; 78.9% boys, 22.1% girls.	M-CHAT; RBS-R; self-developed questionnaire	8.84 hours per day	Screen use was associated with increased severity of ASD symptoms and restricted behaviors. Reduced social interaction correlated with an increase in repetitive behaviors.
Garcia et al, 2023 17	United States	897 ASD patients. Mean age: 14.38 years; 82% boys; 18% girls	Self-developed questionnaire	Mean screen time not reported in the study; however, 49.1% use screens for 2–3 h/day; 21.8% for >4 h/day	Greater screen time was associated with overeating/obesity, reduced academic engagement, and difficulty forming friendships.
Must et al., 2023 18	United States	187 ASD patients. No mean age or sex data reported	YSTS; self-developed questionnaire	Boys: 174 min (passive); 102.7 min (video games); Girls: 180 min (passive); 64.4 min (video games)	Greater screen time was associated with social isolation, sedentary behavior, and obesity.

Abbreviations: ADOS-2, Autism Diagnostic Observation Schedule, 2 ^nd^ Edition; ASD, autism spectrum disorder; CARS, Childhood Autism Rating Scale; CBCL, Child Behaviour Checklist; CSHQ, Children's Sleep Habits Questionnaire; GDS-C, Griffiths Development Scales for China; M-CHAT, Modified Checklist for Autism in Toddlers; RBS-R, Repetitive Behaviors Scale – Revised; YSTS, Youth Screen Time Survey.

While Garcia et al. 9 found no association between screen time and sleep quality in an adolescent ASD population using screens within the recommended limits, 9 an Indian study 10 utilizing validated scales in preschoolers with excessive screen exposure identified associations with delayed sleep onset, shorter sleep duration, increased sleep anxiety, nocturnal awakenings, and daytime sleepiness. 10

In this study, an additional hour of screen time was associated with a 0.216-point increase in sleep onset delay score ( *p* = 0.048), a 0.863-point increase in sleep duration score ( *p* = 0.004), a 0.962-point increase in sleep anxiety score ( *p* = 0.005), a 0.645-point increase in nocturnal awakenings score, and a 1.794-point increase in daytime sleepiness score ( *p* = 0.007). Furthermore, an additional hour of screen exposure correlated with a 5.611-point increase in the Children's Sleep Habits Questionnaire (CSHQ) total score ( *p* = 0.014).

Cardy et al. 11 reported that excessive screen use in older male children was associated with a higher likelihood of negative impacts on quality of life, with an odds ratio (OR) of 1.8 and 95% confidence interval (CI) of 1.1–2.9 ( *p* = 0.040), and mental health, with OR of 1.9 and 95% CI of 1.1–3.1 ( *p* = 0.0028). The perceived negative impact was positively correlated with total screen time, hours spent playing video games, and time spent watching videos. However, time spent connecting with friends and family, using social media, engaging in educational games, and using therapeutic applications was associated with a lower likelihood of perceived negative impact. Additionally, ASD children were 2.7 times more likely to experience social interaction deficits, although the specific nature of these interactions was not detailed. 11

A Chinese study 12 in preschoolers found a negative correlation between screen time and the Griffiths Development Scales for China (GDS-C), particularly in the auditory and speech development domain, and a positive correlation with the Childhood Autism Rating Scale (CARS). This correlation was stronger in children with daily screen exposure exceeding 2 hours. 12 Another Chinese study 13 in a similar population found that ASD children had approximately 2 additional hours of daily screen time compared to neurotypical peers, also reporting negative correlations with GDS-C and positive associations with CARS scores. 13 The negative correlations extended across all GDS-C domains, including adaptive behavior, gross motor skills, fine motor skills, language, and personal-social behavior.

Another Chinese study 14 in preschoolers identified a positive correlation between presleep screen exposure and total scores on the Child Behaviour Checklist (CBCL), indicating increased behavioral problems. The sleep problems and somatic complaints subscales of the checklist also showed positive correlations. Additionally, pre-sleep screen exposure correlated positively with total CSHQ scores, with significant associations in the subscales for sleep-disordered breathing and parasomnias. 14

A French study highlighted age as a critical factor, with excessive screen time being significantly more prevalent among adolescents (63.6% in those aged ≥ 12 years) compared to younger children (30% when < 6-year-olds, and 27.3% in 6–11-year-olds). 15 Excessive screen time was significantly correlated with lower scores on the Vineland Adaptive Behavior Scales (VABS-II), particularly in the daily living skills domain, and higher scores on the CBCL withdrawal subscale. Parents of children with excessive screen time, defined as >1 hour per day for those under 5 years and >2 hours per day for older ones, described them as more socially withdrawn, shy, and less interactive. No significant correlation was found between Autism Diagnostic Observation Schedule, Second Edition (ADOS-2) scores and screen time in children under 12 years. However, in adolescents, univariate analysis suggested a possible association between excessive screen time and higher ADOS-2 scores, indicating increased autism severity.

An Iranian study in preschoolers found a significant positive correlation between daily screen time (fore- and background media exposure) and scores on the Modified Checklist for Autism in Toddlers (M-CHAT). 16 Each additional hour of foreground screen exposure was associated with a 0.38-unit increase in ASD symptom severity, while an additional hour of background screen exposure resulted in a 0.29-unit increase in the same scale. Positive correlations were also observed between foreground and background screen exposure and subscales of the Repetitive Behaviors Scale – Revised (RBS-R), with an additional hour of foreground screen time leading to a 0.30-unit increase in restricted behaviors and a 0.38-unit increase in sameness behaviors. Similarly, an additional hour of background screen time resulted in a 0.30-unit increase in ritualistic behaviors and a 0.41-unit increase in sameness behaviors. Reduced social interaction time was linked to a 0.49-unit increase in stereotyped behaviors, a 0.29-unit increase in self-injurious behaviors, a 0.30-unit increase in sameness behaviors, a 0.28-unit increase in compulsive behaviors, and a 0.36-unit increase in total RBS-R score. 16

## DISCUSSION

The data indicate that the average daily screen time exceeded 2 hours in many of the analyzed samples, with significant rates of passive use, such as watching television. For instance, in one US-based study, the mean screen time was 2.64 hours per day, and 40.9% of children had exposure levels above the recommended limits. Similarly, Chinese studies reported comparable patterns, with averages ranging from 2.64 to 2.85 hours and negative associations between excessive screen time and socioemotional development. This excessive pattern aligns with previous literature suggesting a high percentage of children with autism spectrum disorder (ASD) engaging in excessive screen use. 19

Existing literature also highlights an increased engagement with other screen-based activities, such as video games, among autistic individuals. This aligns with our findings, as one study demonstrated that boys with ASD spent approximately 1 additional hour playing video games compared to their neurotypical peers. 20 Our study also found that older boys had a higher prevalence of excessive screen use, although certain screen activities—such as connecting with family or friends and engaging in educational games—may have positive aspects. 11

All studies that included preschool-aged children employed reliable, validated assessment tools and consistently demonstrated neurodevelopmental impairments associated with excessive screen exposure. Although our participants were, on average, 3-years-old, early screen exposure has been increasingly documented in toddlers. In our practice, it is very common to observe early exposure to screens among infants, highlighting that this phenomenon occurs even earlier than expected.

Additionally, increased screen use was linked to hyperactivity, irritability, aggression, oppositional behavior, attention deficits, and socialization difficulties. Evidence suggests that greater screen exposure is directly associated with reduced modulation of neural connections related to cognitive and attentional control. 21 Neuroimaging studies further demonstrate that excessive screen time impairs functional brain connectivity, leading to delayed language development and increased autism symptom severity. 22

A longitudinal intervention study by Heffler et al. found that reducing screen time resulted in a significant decrease in ASD-related symptoms, as measured by the total score of the Brief Observation of Social Communication Change (BOSCC). 23 Their findings indicated an overall 23% improvement in symptoms following screen time reduction. Additional studies have reported delays in the development of auditory processing, speech, and hand-eye coordination associated with excessive screen use, 24 further supporting the results observed in our analysis.

Regarding sleep disturbances, most studies identified an association between screen use and insomnia, as well as reduced sleep quality and duration. 25 Mazurek et al. 26 highlighted that the use of electronic devices before bedtime exacerbates these disturbances, potentially due to factors such as blue light exposure and circadian rhythm dysregulation, leading to melatonin-related dysfunctions. 26 27 Circadian rhythm delay, which has been described in adolescents with ASD, may also be related to these findings, reinforcing their predisposition to sleep difficulties.

However, Garcia et al. employed objective sleep-tracking devices to quantitatively and qualitatively assess sleep patterns and found no significant associations between screen time and sleep quality in adolescents who averaged 130 minutes of daily screen use—within the WHO's recommended limits. 9 This finding suggests that adherence to screen time guidelines may be appropriate for the ASD population. Nonetheless, another study examining a similar population, even within the recommended screen exposure limits, still identified significant sleep disturbances. 11

Two multicenter studies reported findings that contrast with our results. However, neither study utilized validated questionnaires to assess behavioral or sleep impacts, nor did they evaluate ASD symptoms, limiting their comparability. Li et al. 28 did not identify a statistically significant association between screen time and general health outcomes or quality of life, although their study assessed compliance with screen time guidelines rather than the actual duration of exposure. 28 Similarly, Montenegro et al. 29 found no significant association between increased screen time and worsened sleep patterns, and identified only a minor but significant relationship between it and heightened anxiety. 29

This study has several limitations. Notably, the lack of standardized sample assessments and variability in measurement tools and outcome metrics complicates cross-study comparisons, as many studies utilized nonstandardized, self-developed questionnaires. Additionally, population heterogeneity may influence findings, given that studies from over six countries were analyzed, each with distinct socioeconomic and cultural differences. Furthermore, none of the studies accounted for the substantial phenotypic variability in ASD, the presence of comorbid disorders, or the baseline severity of symptoms—factors that could introduce bias into the results.

In conclusion, the present study demonstrated that excessive screen use among children with autism spectrum disorder (ASD), particularly in preschool-aged children, may have significant behavioral impacts. Given these findings, the WHO's screen time recommendations for the general pediatric population should also be applied to this cohort, with particular caution for younger children, until further research provides specific guidelines tailored to their phenotypic variability and psychiatric comorbidities.

Considering the rising prevalence of ASD and its diverse clinical manifestations—potentially exacerbated by excessive digital screen exposure—it is crucial to develop and disseminate strategies aimed at limiting screen use in both educational and home settings. Further studies are needed to refine these recommendations and assess their long-term implications for neurodevelopment and overall well-being in this population.
